# Case of Poisoning by Laudanum

**Published:** 1860-09

**Authors:** H. Wardner

**Affiliations:** Demonstrator of Anatomy in Lind University


### CASE OF POISONING BY LAUDANUM.—COLD WATER TREATMENT.

By H. WARDNER, M. D.,
Demonstrator of Anatomy in Lind University,

*Case* 1st. Miss Marion ———, in a fit of anger swallowed an ounce of Laudanum, fortunately, in the presence of the family. Happening to pass the house at the moment, I was called in, and administered a full dose of tartarized antimony, within five minutes from the time the Laudanum was taken. A free and prompt emesis followed, expelling it entirely from the stomach. In three hours, she was as well as usual; no doubt, secretly congratulating herself upon giving her friends a fright, and escaping the slightest harm.

*Case* 2d. A Miss B———, of this city, in a violent fit of jealousy, drank between one and two ounces of Laudanum.

Some thirty minutes or more elapsed before I saw her. A large dose of tart. emet., was at once administered, and followed by copious draughts of warm water. Although the opium had not very perceptably effected the brain, yet the emetic was slow to act. Upon pouring cold water on her head, a prompt action took place, and the stomach was relieved of the drug, together with an enormous supper with which she had fortified herself to meet death. There were no subsequent effect worthy of note.

*Case* 3d. In the fall of 1859, I was called to see a girl, who, as was afterwards ascertained, had taken an ounce of Laudanum, about six hours previous. She was found upon the bed by the family, where she stopped, who were unable to arouse her. Her appearance answered the discriptions of opium poisoning. I tried various means for arousing her, in vain, until, after thrusting pins into her hands and feet for a while, I observed some slight twitching of the muscles. She was then taken out of bed; her head held over a tub; and I poured upon the top of it, a stream of cold water from a pitcher three or four feet above her head, for nearly twenty minutes, when she drew a deep, full breath, partly opened her eyes, and shortly after vomited. I kept up the stream of cold water, till she begged me to stop. I then gave her very strong coffee, and shortly had the satisfaction of seeing my patient fully recovered, satisfied with the folly of self destruction.

*Case* 4th. In June last, I was called to see Miss Emily C———, who an hour previous, had taken full two ounces of Laudanum on nearly an empty stomach. Found an "apothecary doctor" with her, who had managed to keep her partly aroused. She had obstinately refused to take anything, and was said to be past swallowing. I prepared a powerful emetic, which she refused, saying indistinctly she "could not swallow it." But *take* it she *must*, and *did*. Her obstinancy being conquered, a deep stupor came speedily on.

The cold water was then used as in case No. 3, after a little delay, emesis occured repeatedly. For five or six hours, she was in a state more or less comatose. Cold water was poured upon her head at intervals of ten to twenty minutes, until she was at each time aroused, so as to answer questions, and beg for mercy.

The effects of the drug at length passed away, and she recovered after a few days of illness, during which there was a strong tendency to dysentery.

This method of treating these cases, has thus proved very satisfactory. It is a means of treatment always at hand, and recommends itself to the public, as well as the profession.

